# Bayesian estimation of a cancer population by capture-recapture with individual capture heterogeneity and small sample

**DOI:** 10.1186/s12874-015-0029-7

**Published:** 2015-04-24

**Authors:** Laurent Bailly, Jean Pierre Daurès, Brigitte Dunais, Christian Pradier

**Affiliations:** Department of Public Health, University Hospital of Nice, Nice, France; Department of Biostatistics, Epidemiology and Clinical Research EA2415, University of Montpellier1, Montpellier, France; Département de Santé Publique, CHU Nice, Hôpital Archet 1. Niveau1, Route Saint Antoine de Ginestière, BP 3079 06202 Nice, Cedex France; IURC - Laboratoire de Biostatistique d’Epidémiologie et de Recherche Clinique, 641 avenue du Doyen G. Giraud, 34093 Montpellier, Cedex France

**Keywords:** Capture-recapture, Cancer population, Capture-recapture models, Bayesian model averaging, Capture heterogeneity, Completeness of cancer registries

## Abstract

**Background:**

Cancer incidence and prevalence estimates are necessary to inform health policy, to predict public health impact and to identify etiological factors. Registers have been used to estimate the number of cancer cases. To be reliable and useful, cancer registry data should be complete. Capture-recapture is a method for estimating the number of cases missed, originally developed in ecology to estimate the size of animal populations. Capture recapture methods in cancer epidemiology involve modelling the overlap between lists of individuals using log-linear models. These models rely on assumption of independence of sources and equal catchability between individuals, unlikely to be satisfied in cancer population as severe cases are more likely to be captured than simple cases.

**Methods:**

To estimate cancer population and completeness of cancer registry, we applied M_th_ models that rely on parameters that influence capture as time of capture (t) and individual heterogeneity (h) and compared results to the ones obtained with classical log-linear models and sample coverage approach. For three sources collecting breast and colorectal cancer cases (Histopathological cancer registry, hospital Multidisciplinary Team Meetings, and cancer screening programmes), individual heterogeneity is suspected in cancer population due to age, gender, screening history or presence of metastases. Individual heterogeneity is hardly analysed as classical log-linear models usually pool it with between-“list” dependence. We applied Bayesian Model Averaging which can be applied with small sample without asymptotic assumption, contrary to the maximum likelihood estimate procedure.

**Results:**

Cancer population estimates were based on the results of the M_h_ model, with an averaged estimate of 803 cases of breast cancer and 521 cases of colorectal cancer. In the log-linear model, estimates were of 791 cases of breast cancer and 527 cases of colorectal cancer according to the retained models (729 and 481 histological cases, respectively).

**Conclusions:**

We applied M_th_ models and Bayesian population estimation to small sample of a cancer population. Advantage of M_th_ models applied to cancer datasets, is the ability to explore individual factors associated with capture heterogeneity, as equal capture probability assumption is unlikely. M_th_ models and Bayesian population estimation are well-suited for capture-recapture in a heterogeneous cancer population.

**Electronic supplementary material:**

The online version of this article (doi:10.1186/s12874-015-0029-7) contains supplementary material, which is available to authorized users.

## Background

Cancer is the leading cause of death in Western countries and particularly in France [1]. In view of this situation, cancer control programmes have been implemented. Evaluating the effectiveness of these policies, aiming for improved prevention and management, is essential. However to conduct such an evaluation, a baseline reference requiring ongoing, reliable and complete data collection, such as a cancer registry, is necessary. Besides providing descriptive epidemiology, cancer registries are currently used for epidemiological research, assessment of screening programmes and treatment innovations [2].

In order to verify the completeness of cancer cases recorded within a specific geographic area, capture-recapture method is usually applied. The capture-recapture method is a sampling technique originally devised by ecologists to study fauna [3,4], and subsequently adapted to epidemiological studies [5-7] Since the early nineties, this method has been extended to many demographic and epidemiologic studies [8,9]. It has thus been used to confirm the completeness of the data recorded in cancer registries [10-12].

The capture-recapture procedure in epidemiology consists in confronting data from at least two independent sources, collecting cases in the same area in order to estimate the total number of cases, and assessing the completeness of each data source [13]. In brief, this method involves modelling the overlap between two or more lists of individuals (data “sources”) from the target population, and using this model to predict how many additional individuals were unseen, and hence the total population size. To avoid bias in the estimate, the sources of data collection must be independent and homogeneity of capture must be ensured [14,15] (i.e. the probability of capture does not depend on case characteristics). Capture heterogeneity can result in positive dependence (underestimation of the population) or negative dependence (over-estimation of the population) between sources.

The standard approach to capture-recapture in epidemiology is to fit log-linear models [16,17], in which the inclusion of source by source interaction terms may account for dependencies between the data sources. These parameters are subsequently estimated by the maximum likelihood estimate procedure. With a sufficiently large population, this procedure is acceptable under the asymptotic assumption. However, the asymptotic assumption cannot be verified on few cases, a frequent situation when capture-recapture concerns a specific target population, as a cancer population within a small area.

Many authors have presented capture-recapture methods in epidemiology to take into account dependence and individual heterogeneity, including source by source interaction terms in the log-linear model, log-linear models stratified on several covariates, or including sources by covariate interactions in a single log-linear model [6,9,18].

In the log-linear method, capture heterogeneity is pooled with between-“list” dependence within the between-source interaction terms [19]. To test capture heterogeneity stratification of cases on potential variables, related to capture heterogeneity, is applied. For example, this method has been applied by King et al.[20] to estimate current injectors in Scotland and drug-related death rate by sex, region and age-group. This consists in constructing a single contingency table in which cells correspond to the numbers of individuals belonging to each distinct combination of covariates and sources.

Conversely, incorporating one or more potential variables is more complex when numbers of cases are limited. Stratification of cases, whether common or not to both sources, on several covariates leads to a contingency table containing several missing cells or too few cases within certain cells to provide robust results.

Moreover, many authors, e.g. Schmidtmann [21] as Silcoks and Robinson [22], compared several methods to estimate the completeness of cancer registration, among which log-linear models: according to both these authors, log-linear modelling does not always yield the best estimation. Confirmation of results obtained via the classical log-linear model therefore appeared to us as essential. Capture heterogeneity, which had not been previously tested with log-linear models, needed to be taken into account. During the past years, much theoretical research has been conducted to develop capture recapture methods, such as that by Chao, Pan and Chiang [23] who propose a Lincoln-Petersen estimator including dependence effects resulting from local lists and heterogeneous capture probabilities. Other authors have proposed mixed models: Mao [24] focused on a non-parametric maximum likelihood estimate for two classes of mixed models with a binomial and geometric distribution. The classical modelling approach consists in estimating the parameters of a model which are then considered as fixed quantities. To confirm our results with a totally different approach, we therefore wished to implement a Bayesian procedure. Several authors have focused on this method, among them Manrique-Vallier and Fienberg [25] who postulated the existence of a homogeneous population class to overcome the problems related to heterogeneity of closed populations in capture recapture.

However, capture-recapture was first developed in ecology for estimating the size of animal populations: as a result, methods in ecology are somewhat more developed and there is probably much for epidemiologists to learn from the ecology literature.

In this paper, we borrow tools from the ecological capture-recapture literature: M_tbh_ models [26], which simultaneously allow for the effects of time, behaviour and individual heterogeneity in capture probabilities. King and Brooks [27], proposed a Bayesian estimate for the size of a closed population in the context of heterogeneity and model uncertainty, using Bayesian Model Averaging (BMA). This approach overcomes the difficulties related to capture heterogeneity and model selection, providing the ecological models may be adapted to capture recapture procedures in epidemiology.

We applied these tools to a capture recapture study concerning a histopathological cancer registry [28]. This study confronted newly diagnosed cases of breast and colorectal cancer, in the Alpes-Maritimes (Southeastern France), among patients aged 50 to 75 years, recorded in the Histopathological Registry (HR), those discussed in hospital Multidisciplinary Team Meetings (MTM) and those diagnosed through the coordinated Cancer Screening Programmes (CSP). We compared the results to those obtained with log linear models and sample coverage approach [19] for the same data [28]. We have then applied ecological models and BMA method to well-known examples of data set in capture-recapture, as an outbreak of the hepatitis A virus in a college in northern Taiwan [29], a data set on diabetes in a community in Italy based on four records [30] and finally to five lists of infants born with a specific congenital anomaly in Massachusetts [31,32].

## Methods

### Capture-recapture ecological models

When estimating a population size using the capture recapture method in an ecological study, the underlying assumptions concerning case capture have a direct impact. The selected model to estimate the total number of cases rests on these assumptions and on its adjustment on the observed data. Otis *et al.* [26] defined three effects influencing capture: time, behaviour and individual heterogeneity. All the interactions may be possible between these three effects. In other words, models differ according to whether the capture probability changes only with time of capture (M_t_), or changes between individuals according to their behaviour (M_b_) or their characteristics (M_h_).

The use of behavioral models (M_b_ and more complex models including the behavioral effect) do not appear appropriate in epidemiology as capture probability should not change after a previous capture. Indeed, they are based on the assumption of a natural sequence of capture episodes, whereas there is no time sequence in our sources so that these models do not appear useful. This has also been pointed out by Chao *et al.* [19] who went as far as stating that only M_t_, M_h_, and M_th_ are potentially useful for applications in epidemiology. For our study, we will therefore focus on these three models applied to our data, implying that capture probability changes only with time of capture (M_t_), or according to individuals’ characteristics (M_h_) or both (M_th_).

Let p_iτ_ denote the capture probability for individual i = 1, 2, 3, …N at time τ = 1, 2, 3, … T. *F*(i) represents the first time that individual *i* is observed. Therefore p_iτ_ = P which is the initial capture probability of *i* for times τ =1,…, *F*(i), and the recapture probabilities for τ = *F*(i) + 1, …T assuming *F*(i) < T. Thus individual *i* is captured at time τ =1, not captured at time τ = *F*(i) – 1 and captured at time τ = *F*(i), and can be recaptured between times τ = *F*(i) + 1 and τ, with total capture times = T.

The saturated *M*_*th*_ model integrating time (t), and heterogeneity (h) has capture probabilities of the form:

$$ \mathrm{Logit}\left({\mathrm{p}}_{i\tau}\right)=\mu +{\alpha}_{\tau }+{\gamma}_i $$

where γ_*i*_ denotes independent and identically distributed individual random effects (i*.*i*.*d) ~ *N* (0, σ^2^_γ_).

In this model the estimated parameters are μ (mean capture rate expressed as logit), α_τ_ (year effect for capture), and σ^2^_γ_ (variance of individual random effects). Submodels of the saturated model are obtained by setting certain parameter values equal to zero.

It is assumed that capture probabilities are independent, given the parameter values. Let θ = {μ, α, σ^2^_γ_, N} with α = {α_1_,…,α_T_}, and γ = {γ_1_,…, γ_N_}. The vector x, which describes the capture history of all individuals, is given by:

1 $$ f\left(\left.x\right|\theta, \gamma \right)\propto \frac{N!}{\left(N-n\right)!}{\displaystyle \prod_{t=1}^T{\displaystyle \prod_{i=1}^N{p_{it}}^{x_{it}}{\left(1-{p}_{it}\right)}^{\left(1-{x}_{it}\right)}}} $$

where *n* (n < N) denotes the total number of individuals captured in the study, i.e. *n* unique individuals captured initially. The vector x_it_ = 0 if individual *i* is not captured at time t and x_it_ = 1 if the individual is captured at time t, i.e. x = (x_11_, …, x_1T_, … x_21_, …, x_2T_, x_N1_, …, x_NT_) where x_it_ = 0 or x_it_ =1.

For models with no heterogeneity, i.e. *M*_t_, the individual random effect γ_i_ = 0 so f(x | θ, γ) = f(x | θ) and θ can be obtained using the Maximum Likelihood Estimate (MLE) of the parameters. In the presence of heterogeneity (models *M*_h_, *M*_th_), calculating the MLE and selecting the model is more complex [33] because of the individual random effects.

### Bayesian population estimation

In the Bayesian approach, individual heterogeneity is estimated from Monte Carlo Markov Chain algorithms. All the parameters can thus be estimated for all possible models, with or without individual heterogeneity. In the Bayesian approach, the model parameters are considered as a random sample. The distribution of the samples therefore provides information on the parameters. Before collecting the data, the parameter distribution is a prior distribution. After data collection, the parameters have a posterior distribution.

In this analysis, the model itself is considered as an unknown parameter to be estimated. According to Bayes’ theorem applied to continuous distributions, the joint posterior distribution over both parameter and model space is obtained by multiplying the likelihood by the prior distribution, with *m* denoting the model and *θ*_*m*_ the parameters in model *m*:

$$ \pi \left({\theta}_m,m\left|x\right.\right)\propto g\left(x\left|{\theta}_m,m\right.\right)p\left({\theta}_m\left|m\right.\right)p(m) $$

To introduce individual heterogeneity, random effects are included as expressed by the variables γ = {γ_1_,…, γ_N_} in equation Eq. 1.

$$ \pi \left({\theta}_m,\gamma, m\left|x\right.\right)\propto f\left(x\left|{\theta}_m\right.\gamma, m\right)h\left(\gamma \left|{\theta}_m\right.\right)p\left({\theta}_m\left|m\right.\right)p(m) $$

The term h(γ|θ_m_) corresponds to the model assumption of the random effect γ_*i*_ ~ *N* (0, σ^2^_γ_).

Finally, the posterior distribution of the parameters and the model is given by:

$$ \pi \left({\theta}_m,m\left|x\right.\right)={\displaystyle \int \pi \left({\theta}_m,\gamma, m\left|x\right.\right)d\gamma } $$

Models are compared via posterior model probabilities and an estimate of the total population, based on all plausible models, may be obtained using the posterior distribution. In other words, each estimate is an average of the posterior distributions under each of the models considered, weighted by their posterior model probability. This procedure, detailed in Additional file 1: Appendix A, is called Bayesian Model Averaging. Usually, a single model is selected, as this model best fits the observed data. However these data come from random sample. As Hoeting pointed out [34] this approach ignores the uncertainty in model selection, leading to over-confident inferences and decisions that are more risky than one thinks they are. Bayesian model averaging (BMA) provides a coherent mechanism for accounting for model uncertainty.

Bayes’ theorem is used to estimate the joint posterior distribution of all the parameters included in the model. Ultimately, the posterior marginal distribution of the parameters of interest is estimated, requiring integration of the posterior joint distribution, which is not always possible. In the modern Bayesian approach, this distribution of the posterior parameter vector is not integrated, and specific simulations are performed to obtain posterior distribution samples and thus simulated values for the posterior marginal distributions of the parameters of interest. As the posterior distribution is multidimensional, Monte Carlo Markov Chain algorithm is applied to obtain a random sample of the posterior distribution. Let’s firstly consider the two components of this method: Monte Carlo integration and Markov chains. Monte Carlo integration is a simulation technique which allows obtaining an estimate of a given integral. This technique is based upon drawing observations from the distribution of the variable of interest and then calculating the sample mean [35]. To obtain a potentially large sample from the posterior distribution we use a Markov chain, which is a stochastic sequence of numbers where each value in the sequence depends only upon the last. Under conditions as chain is aperiodic and irreducible, distribution will converge to a stationary distribution. Monte Carlo Markov Chain methods perform Markov chains with Monte Carlo integration to generate observations and to construct a sequence of values whose distribution converges to the posterior distribution of interest. Once the chain has converged we can use sequence of values to obtain estimates of any posterior summaries of interest (Monte Carlo). To be sure that Markov chain has reached the stationary distribution before Monte Carlo estimates, we discard observations from the start of the chain, which is called the burn-in.

Two Monte Carlo Markov Chain algorithms are used according to the parameters and models. The Metropolis-Hastings algorithm is used when the model does not involve changing the dimension and the reversible jump algorithm when calculations involve a change in dimension (due to the model and the population size in the presence of individual effects). The reversible jump algorithm is detailed in Additional file 1: Appendix B.

Lastly, prior probabilities must be defined for the models and parameters. In the absence of prior assumptions concerning their influence on the estimate of the total population, we chose a non-informative model. The priors for each parameter are detailed in Additional file 1: Appendix C. The prior probability of selecting a model follows a uniform distribution p(m) = 1/k where k denotes the number of models. For each potential effect (time, heterogeneity) the prior probability is 0.5. The parameters are assumed to be independent and to follow the same prior distribution in each model (if present).

### Software for Bayesian analysis

We applied the above Bayesian methods using the WinBUGS [36] software package, which performs complex Bayesian analyses. The codes used for the M_th_ models were drawn from the models developed by Link and Barker [37] in WinBUGS for Bayesian inference applied to ecological surveys, namely capture recapture. Briefly, two vectors are used: the number of captures of each individual and the fact that an individual is considered as caught, not caught or unknown during a specific episode. To provide an inference for the total population we consider that the individual capture probabilities of the subjects that were not caught are linked to the capture probabilities of those that were captured. The total number of subjects is estimated by the method of data augmentation developed by Link and Barker*.* The WinBUGS codes are detailed in Additional file 1: Appendix D.

### Individual covariates

The three sources studied are the Histopathological Registry, the hospital Multidisciplinary Team Meetings (MTM) and the Cancer Screening Programmes. Firstly, histopathology laboratories have been transmitting all the invasive cancers with a histopathology diagnosis to the Nice University Hospital Public Health Department, which coordinates the Histopathological registry since 2005. Cancer cases from hospital Multidisciplinary Team Meetings came from the regional cancer network which has been systematically collecting data from hospital since 2007. The third source is the local coordinating centre for cancer screening which collects data concerning patients aged 50 to 75 years with a positive result following screening for breast or colorectal cancer since 2002 and 2005 respectively.

An estimate was first obtained from the three available sources, each of them considered as a capture episode. Secondly, an estimate of the total population was obtained by considering each covariate as a capture episode. The selected parameters considered as potentially accounting for different capture probabilities included age and presence of metastases at the time of diagnosis (TNM staging), according to the recommendations concerning capture recapture applied to cancer registries [38,39]. We also introduced past history of screening mammography [21] and gender for cases of breast and colorectal cancer, respectively, as potential capture heterogeneity parameters.

## Result

### Estimate of the number of incident cases of breast cancer

**Figure 1:**
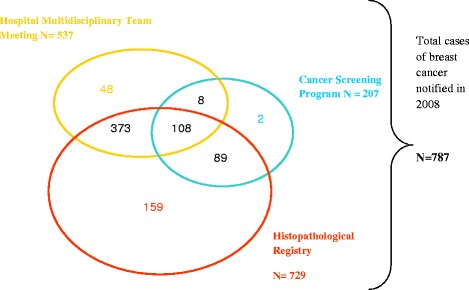
Record linkage of breast cancer cases notified in 2008 among subjects aged 50 to 75.

Capture-recapture M_th_ models were initially applied to the three sources, each considered as a capture episode, i.e. Histopathological cancer Registry (HR), hospital Multidisciplinary Team Meetings (MTM) and cancer screened cases (CSP). In these 3 sources, 787 cases aged 50 to 75 years were notified in 2008 as presented in Figure 1, of which 729 by the Histopathological cancer registry, 470 were identified through at least 2 sources and 108 were common to all three sources. After averaging over all the models according to their posterior probability, the estimated mean number of breast cancer cases was 790.6 (median = 790.2, 95%HPDI: 790.2-792.2), as presented in Table 1. The average estimate was obtained over all models following 25 000 iterations after discarding the initial 5 000 iterations. Since first iterations a convergence of Markov chains was obtained, as a stationary distribution was observed after only 500 iterations.

**Table 1:** **Estimates of incident breast cancer cases according to capture-recapture M**_**th**_ **models 3 capture episodes: Histopathological Registry, hospital Multidisciplinary Team Meeting, and Cancer Screening Program**

**Model**	N*	Sd**	**Posterior probabilities**	**Median**	**95%HPDI**
M_t_	790.4	0.5	100.0%	790.2	790.2-791.2
M_h_	814.1	4.9	-	814.2	805.2-824.2
M_th_	806.3	4.5	-	806.2	798.2-815.2
**M**_**avg**_ ***	**790.6**	0.63		790.2	790.2-792.2

*Estimated total number of cases **Standard Deviation ***Average estimate over all models following 25 000 iterations after discarding the initial 5 000 iterations.

With a total number of 791 breast cancer cases, the completeness of the Histological Register was estimated to be 92.2% (92.1%; 92.3%). The posterior probability for the M_t_ model was 100%, corresponding to the model for which capture probability changes for each source. As there is no time sequence in our sources, we altered it and compared the estimates obtained for each possible time sequence. Estimates were exactly the same for all possible time sequences. Models for which the capture probability differs for each individual (M_th_ M_th_) provide a different estimate: 814.1 (median = 814.2, 95%HPDI: 805.2-824.2) and 806.3 (median = 806.2, 95%HPDI: 798.2-815.2) cases, respectively.

Averaging over the M_th_ models, applied to the three sources, provides an estimate in line with the result obtained by the classical method [28]. With the selected log-linear model, the estimate was of 791 breast cancer cases corresponding to the model including interaction between the Multidisciplinary Team Meeting source and the two other sources, as presented in Table 2. The choice of the most appropriate log-linear model is based on the likelihood ratio statistics. The selected model is the one with the fewest interaction terms and the best fit with the observed data, i.e. a non-significant value for the likelihood ratio statistics. The model was selected using a stepwise descending procedure starting from the saturated model, taking all interactions into account, until the most parsimonious model with the best fit was retained. The total number of cases was estimated to be 791 cases (*95% CI: 784-797)*, i.e. the completeness of the histological register was estimated to be 92.2% (*95% CI: 91.5%; 93.0%)*. Taking into account coefficient of covariation as developed by Chao et al. [19], the sample coverage approach gives an estimate of 794 cases CI 95% [788-824] with dependent sources and an estimate of 802 cases CI 95% [795-815] with three independent sources, close to the estimates obtained previously.

**Table 2:** **Estimated total number of incident breast cancer cases according to log-linear models Histopathological Registry (HR), hospital Multidisciplinary Team Meeting (MTM), and Cancer Screening Program (CSP)**

***Models***	N*	***95% CI***	***df***	G2**	***P***
θ + HR + MTM + CSP + HR*MTM + HR*CSP + MTM*CSP	793	[782–804]	0	0	1,0
θ + HR + MTM + CSP + HR*MTM +HR*CSP	799	[779–819]	1	14,6	<0,001
θ + HR + MTM + CSP + MTM*CSP + HR*CSP	807	[796–819]	1	2,7	0,10
θ + HR + MTM + CSP + MTM*CSP + HR*MTM	**791**	**[784–797]**	**1**	**2,2**	**0,15**
θ + HR + MTM + CSP + MTM*CSP	803	[794–813]	2	10,6	0,01
θ + HR + MTM + CSP + HR*CSP	812	[799–824]	2	15,6	<0,01
θ + HR + MTM + CSP + HR*MTM	793	[783–802]	2	19,5	<0,01
θ + HR + MTM + CSP	806	[793–820]	3	24,6	<0,01

*Estimation by model of the total number of breast cancer cases **G2 = Goodness-of-fit for nested models G2M1 - G2M2.

### Dependence between sources

For breast cancer cases, the Lincoln-Petersen estimate [28] obtained via two-by-two record linkage for the MTM and CSP sources (N_MTM-CSP_ = 958) differed from the estimates obtained from the other sources (N_HR-MTM_ = 814 and N_HR-CSP_ =766). Interdependency between these two sources was suspected and confirmed by a test for independence [6] on the basis of whether cases were recorded or not in the third source (OR_MTM-CSP_ = 0.52; 95% CI:0.37-0.73). If interdependence is shown between two of at least three data sources, these must be pooled in order to apply the capture recapture procedure to two independent sources. The resulting Lincoln-Petersen estimate, by cross-linkage of Histopathological Registry cases and cases discussed during MTM pooled with screened cases, was N = 803.2 (95% CI: 793.8-812.5).

### Capture heterogeneity

To investigate capture heterogeneity between individuals with breast cancer, we created 21 capture episodes, based on potential heterogeneity covariate: age, expressed as 5-year intervals, i.e. five capture episodes for each of the three sources, presence of metastases at the time of diagnosis and, finally, history of screening mammography by sources, i.e 6 capture episodes. The estimate averaged over all the models was of 803 cases (median = 802.6, 95%HPDI: 798.6-809.6), based on the results of the M_h_ and M_t_ models with 80% and 20% of posterior probability, respectively, as presented in Table 3. The average estimate was obtained over all models following 25 000 iterations after discarding the initial 10 000 iterations. Convergence of Markov chain to a stationary distribution was observed after 10 000 iterations.

**Table 3:** **Estimates of incident breast cancer cases according to capture-recapture M**_**th**_ **models 21 capture episodes: age, TNM stage at diagnosis, screening history**

**Model**	N*	Sd**	**Posterior probabilities**	**Median**	**95%HPDI**
M_t_	797.6	0.1	20.0%	797.6	797.6-797.6
M_h_	802.8	2.8	80.0%	802.6	798.6-808.6
M_th_	803.7	2.9	-	803.6	798.6-810.6
**M**_**avg**_ ***	**803.0**	2.8		802.6	798.6-809.6

*Estimated total number of cases **Standard Deviation ***Average estimate over all models following 25 000 iterations after discarding the initial 10 000 iterations.

This result is in line with the Lincoln-Petersen estimate for pooled MTM and cancer screening sources. Therefore, the estimated completeness of the Histopathological Registry for breast cancers would be of 90.8% (90.0%-91.3%).

### Estimate of the number of incident cases of colorectal cancer

**Figure 2:**
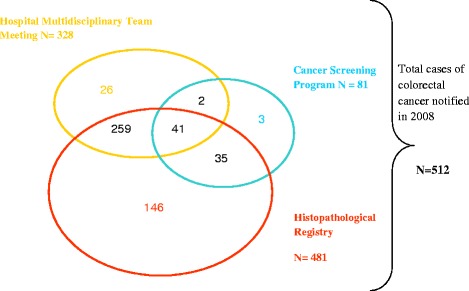
Record linkage of colorectal cancer cases notified in 2008 among subjects aged 50 to 75.

Results from the three sources showed 512 cases of colorectal cancer in 2008 (HR, MTM, CSP), of which 481 were recorded in the Histopathological Registry, 337 were identified through at least 2 sources and 41 were common to all three sources as shown in Figure 2.

Using the BMA method, ecological M_th_ models were first applied to the three sources collecting incident colorectal cancer cases, considered each as a capture episode. The estimate averaged over all the models yielded 513 cases (median = 513.0; 95% HPDI: 512.-515.0) based on the results of the M_t_ model, as presented in Table 4. Estimates were exactly the same for all possible time sequences. The average estimate was obtained over all models following 25 000 iterations after discarding the initial 5 000 iterations. As for breast cancer, convergence of Markov chains to a stationary distribution was observed from the beginning, meaning before 1000 iterations.

**Table 4:** **Estimates of incident colorectal cancer cases according to capture-recapture M**_**th**_ **models 3 capture episodes: Histopathological Registry, hospital Multidisciplinary Team Meeting, and Cancer Screening Program**

**Model**	N*	Sd**	**Posterior probabilities**	**Median**	**95%HPDI**
M_t_	513.0	1.9	98.8%	514.6	511.6-519.6
M_h_	532.0	4.6	1.0%	532.0	523.0-541.0
M_th_	526.5	4.2	0.2%	526.0	519.0-535.0
**M**_**avg**_ ***	**513.0**	1.6		513.0	512.0-515.0

*Estimated total number of cases **Standard Deviation ***Average estimate over all models following 25 000 iterations after discarding the initial 5 000 iterations.

With a total number of 513 colorectal cancer cases, the completeness of the Histological Registry was estimated to be 93.8% (93.4%; 94.0%). With the selected log-linear model [28], the estimate was of 527 colorectal cancer cases corresponding to the model without interaction, as presented in Table 5. With sample coverage approach, estimates are 527 cases CI 95% [519-542] with three independent sources, as selected log-linear model without interaction, and 529 cases CI 95% [519-557] with dependent sources.

**Table 5:** **Estimated total number of new cases of colorectal cancer according to log-linear models Histopathological Registry (HR), hospital Multidisciplinary Team Meeting (MTM), and Cancer Screening Program (CSP)**

***Models***	N*	***95% CI***	***df***	G2**	***P***
θ + HR + MTM + CSP + HR*MTM + HR*CSP + MTM*CSP	513	[510–516]	0	0	1,0
θ + HR + MTM + CSP + HR*MTM +HR*CSP	551	[479–623]	1	2,7	0,1
θ + HR + MTM + CSP + MTM*CSP + HR*CSP	527	[517–536]	1	0,4	0,6
θ + HR + MTM + CSP + MTM*CSP + HR*MTM	525	[501–548]	1	1,1	0,3
θ + HR + MTM + CSP + MTM*CSP	526	[516–535]	2	1,1	0,6
θ + HR + MTM + CSP + HR*CSP	528	[518–538]	2	3,7	0,2
θ + HR + MTM + CSP + HR*MTM	529	[508–549]	2	4,5	0,1
**θ + HR + MTM + CSP**	**527**	**[517–536]**	**3**	**4,6**	**0,2**

*Estimation by model of the total number of breast cancer **G2 = Goodness-of-fit for nested models G2M1 - G2M2.

### Dependence between sources

As for breast cancer cases, two-by-two record linkage using the Lincoln-Petersen estimator for MTM and CSP sources provided a different result from the two other estimates (N_MTM-CSP_ = 618 versus N_HR-MTM_ = 526 and N_HR-CSP_ =513). However, testing for independence gave a statistically non-significant result (OR = 0.66 [0.40-1.08]). The Lincoln-Petersen estimator [28], for the Histopathological Registry and pooled MTM and CSP sources, yielded an estimate of 525 cases (95% CI: 516.5-534.5).

### Capture heterogeneity

Capture heterogeneity among the colorectal cancer cases was also investigated. Retained covariates potentially responsible for heterogeneity included age in 5 year intervals (15 capture episodes), gender (6 capture episodes) and metastases present at the time of diagnosis, i.e. 15, 6 and 3 capture episodes respectively and 24 for all three sources. Contrary to the results obtained with 3 sources, the estimate, averaged over all the models, was of 521 cases (median = 520.6; 95%HPDI: 517.6-526.6), resulting from the M_h_ model including individual capture heterogeneity, with a posterior probability of 99%, as presented in Table 6. The average estimate was obtained over all models following 25 000 iterations after discarding the initial 5 000 iterations. Contrary to breast cancer with potential heterogeneity covariate, for colorectal cancer convergence of Markov chains to a stationary distribution was observed rapidly after 1 000 iterations. The estimated completeness of the Histopathological Registry would be of 92.3% (91.3%-92.9%).

**Table 6:** **Estimates of incident colorectal cancer cases according to the capture-recapture M**_**th**_ **models 24 capture episodes: age, TNM stage at diagnosis, gender**

**Model**	N*	Sd**	**Posterior probabilities**	**Median**	**95%HPDI**
M_t_	517.6	0.0	1.0%	517.6	517.6-517.6
M_h_	521.0	2.5	99.0%	520.6	517.6-526.6
M_th_	521.0	2.4		520.6	517.6-526.6
**M**_**avg**_ ***	**521.0**	2.5		520.6	517.6-526.6

*Estimated total number of cases **Standard Deviation ***Average estimate over all models following 25 000 iterations after discarding the initial 5 000 iterations.

### Application to data set from other fields

Then we apply our method to an outbreak of the hepatitis A virus in a college in northern Taiwan [29] with 271 cases ascertained by three sources, to a data set on diabetes in a community in Italy based on four records [30] with 2069 cases identified and finally to fives lists of 537 infants born with a specific congenital anomaly in Massachusetts [31,32]. For the HAV data, our method gives an estimate of 515 cases [465.5-567.5] whereas one-step estimator by sample coverage approach gives 508 cases [442-600], Petersen estimator 336 cases and log-linear models 1300 cases. The number of HAV infected students was finally known with a screen serum test for HAV antibody for all students and was about 545.

For the data set on diabetes, author found that the selected log-linear model that fits data gave an estimate of 2 771 cases but they further stratified for heterogeneity terms and an estimate of 2 586 cases [2341-2830] was obtained. With sample coverage approach Chao *et al.* estimate was 2 559 cases [2472-2792] and with our method estimate is 2 589 cases [2534-2645].

For the data set on infants’ congenital anomaly, Wittes and Fienberg [31,32] obtained a close estimate respectively 638 cases under independent assumption and 634 cases for the log-linear model approach. For the sample coverage approach, the retained estimator with dependencies was 659 cases [607-750]. With our method estimate is close to previous ones as it is 654 cases [631-680].

## Discussion

Log-linear models provide a useful method to estimate population size but some authors have pointed out [19,38], the need to pursue additional methods because of assumptions, as independence of sources and equality of capture probability, which are rarely satisfied. Capture-recapture M_th_ models are interesting for cancer population as individual capture heterogeneity is taken into account. Bayesian population estimation allows small sample as it does not rely on the asymptotic assumption.

Firstly, we applied capture-recapture M_th_ models to epidemiological data. Secondly, we applied a Bayesian Model Averaging (BMA) method to present a result averaged over all the models. The BMA method was of interest to us because it takes into account all possible models instead of selecting only the result of the best model. However, we chose to apply capture-recapture M_th_ models specifically for this study because individual heterogeneity was suspected between severe cancer cases collected via Multidisciplinary Team Meetings (MTM), and simple cancer cases screened in a Cancer Screening Program (CSP). These methods can be used separately and this is what we have done in our study. The results for each model were analysed and the BMA method was then applied to obtain a result weighted for all models according to their posterior probability.

For both types of capture-recapture M_th_ models, the samples are independent only for the M_t_ model, while heterogeneity arises for the M_h_ model. The Rasch-like model is the M_th_ model which extends the M_h_ model by allowing time effects. Heterogeneity between individuals means that even if two lists are independent within individuals, the two sources may become dependent if the capture probabilities are heterogeneous among individuals. Model M_h_ assumes that each individual has its own unique probability that remains constant over samples. Capture-recapture M_th_ models have already been used in the context of lists. Chao [19] for example has shown that results of models M_h_ and M_th_ were very close to those obtained with log-linear models by Wittes [31] for 5 lists of “Infants’ congenital anomaly data”. Chao’s conclusion was that although heterogeneous models did not consider possible local dependence, the estimates were close to the proposed estimate that does. We came to the same conclusion in our study comparing estimates yielded by capture-recapture M_th_ models with results obtained by the “source pooling” method. When two of at least three data sources are dependent, these must be pooled in order to apply the capture recapture procedure to two independent sources. “Source pooling” is a method proposed by Wittes [6] and adopted by many authors thereafter. It was applied in this study for comparison with previously published results on these data [28]. We presented this method here to emphasize that the results are in line with the classical methods (log-linear model on three sources or pooled if dependent) and capture-recapture M_th_ models. The interest of these models is the ability to decide to “capture’ subjects aged 60 to 65 years, or diagnosed with metastases, or any others covariates suspected for heterogeneity, whereas with the log-linear approach, the number of potentially adequate models increases and model selection is more difficult.

Finally, capture-recapture M_th_ models allowed us to compare and to confirm results obtained with log-linear models and then to make them more reliable. This last point was particularly important as heterogeneity in three-list data could involve that our estimates were not reliable [14]. Moreover the results retained with the log-linear approach were the estimates of the selected model whereas other fitted models could have yielded a quite different estimate [40].

The objective of this article was to propose an alternative to the method most often used, i.e a log-linear model stratified on several covariates. For example, Tilling [18] did not propose a log-linear model but a logit model which has the advantage over the log-linear model stratified on several covariates to limit the number of parameters studied, to incorporate continuous covariates and above all of being applicable to two sources. However, the adjustment used the maximum likelihood ratio based on the asymptotic assumption, which cannot be verified in our case due to the small number of cases, which is frequent in epidemiology.

Considering each of the three sources (HR, MTM and CSP) as a capture episode, the estimated mean number of breast cancer cases was 790 and the number of colorectal cancer cases 513, according to the M_t_ model. When considering each covariate as a capture episode, the model retained in BMA corresponds to the model with heterogeneity. The estimated total number of cases, for breast cancer, was of 803 cases according to the M_h_ model against 791 cases for the log-linear model [28]. The resulting Lincoln-Petersen estimate from the source-pooling method was of 803 cases too and sample coverage approach gives estimates of 794 and 802 cases, respectively with dependent and independent sources. From our point of view, the estimate from the M_h_ model could be considered as more representative than the results of all the log-linear models considered for breast cancer. Finally, the discrepancy, without considering heterogeneity, between estimates may seem not apparent. However, we have shown with some covariates, corresponding to our heterogeneous population, that heterogeneity exists and has an impact. Indeed, as there were very few cases missing, the difference is equal to two points for histological cancer registry completeness.

The only difference between the M_t_ and M_h_ models lies in the selected covariates considered each as a capture episode which could influence the probability of capture for each individual case. Size effects due to smaller samples cannot be held responsible for a higher posterior probability for model M_h_ because the total number of cases and the gap between samples are the same with 3 episodes of capture. Furthermore we modified the time sequence of our sources, as there is no sequential time order in lists of individuals, and estimates were the same. For colorectal cancer, the estimate of 521 cases for the capture-recapture M_h_ model was in line with the estimated total number of 527 cases retained by the selected log-linear model and by sample coverage approach. The Lincoln-Petersen estimator, for HR and pooled MTM and CSP sources, yielded an estimate of 525 cases.

Application of capture-recapture M_th_ models have confirmed estimates obtained via the log-linear models retained according to the traditional procedure. The traditional model selection procedure and the use of the capture-recapture M_th_ models thus yield concordant results.

For our study, a major advantage of this Bayesian population estimation was the possibility of easily taking into account several covariates potentially responsible for capture heterogeneity, even with few cancer cases collected by some sources. Considering some potential heterogeneity covariates (i.e. age, presence of metastases, screening history or gender) as a capture episode has shown that capture probability was not homogenous between individuals. This can be easily understood for our heterogeneous cancer population as a case with metastases at the time of diagnosis won’t have the same capture probability as other cases, since multidisciplinary team meetings will be more concerned with such situations of advanced disease, whereas there are fewer cases with metastases in a cancer screening program.

However, log-linear method, sample coverage approach and capture-recapture M_th_ models have their advantages and their limitations, which is in favour of applying them both to make estimates more reliable. The main advantage of the log-linear method is that it is particularly well suited to the so-called « list » method. All models have the same framework, the selected model can be tested easily, between-source dependencies are included in interaction terms and inference is easily available in statistical software.

Applying the Bayesian procedure to the M_th_ capture recapture models has the advantage of taking case-linked capture heterogeneity into account and providing a result that incorporates all the possible models. Furthermore, with the Bayesian method, considering a potential heterogeneity covariate as a capture episode may be easily applied to small samples, which can be particularly useful in cancer epidemiological studies. On the other hand, covariate selection may seem arbitrary, which is in favour of selecting variables that have already been shown to have an impact on capture probability [18,39,21]. Bayesian inference is nowadays easily available with the WinBUGS software package [36]. Moreover, codes for M_th_ models and Bayesian Model Averaging have been developed [37] by ecological researchers and can be adapted to epidemiological data.

## Conclusions

Our analysis shows that capture-recapture M_th_ models can be applied to data usually available as « lists » in epidemiology. The advantage of these models resides in their capacity to independently assess heterogeneity of individual capture probability which is useful for a heterogeneous cancer population. Moreover, Bayesian population estimation allows including several covariates potentially accounting for heterogeneity even with small sample. Thus, capture-recapture M_th_ models and Bayesian population estimation should be considered additionally to the classical methods usually implemented in cancer epidemiology, to confirm results and enhance the reliability of estimates.

### Availability and requirements

Winbugs software is available through: http://www.mrc-bsu.cam.ac.uk/software/bugs/the-bugs-project-winbugs.

## Additional file

Additional file 1:
**Appendices.** A) Bayesian Model Averaging. B) Reversible jump MCMC model. C) Prior for the total population. D) WinBUGS Codes.

## Abbreviations

HR: Histopathological Registry
MT: Multidisciplinary team meetings
CSP: Cancer screening programmes
