# Supplementary material for: Bayesian estimation of a cancer population by capture-recapture with individual capture heterogeneity and small sample
Source: BMC Med Res Methodol. 2015 Apr 24;15:39. doi: 10.1186/s12874-015-0029-7 (PMC4421924; doi:10.1186/s12874-015-0029-7)
Supplement: Additional file 1: — Appendices. A) Bayesian Model Averaging. B) Reversible jump MCMC model. C) Prior for the total population. D) WinBUGS Codes. [file 12874_2015_29_MOESM1_ESM.pdf]

**Appendix:**

**A. Bayesian Model Averaging**

With the traditional approach, a single model among the possible candidates is usually selected, so that the decision rests upon that model. However this implies ignoring the uncertainty linked to the selection of a particular model, leading to an unstable result. Such instability is a consequence of the choice of sample which is necessarily random. To integrate uncertainty, it has been suggested to average over all the competing models [42]. This approach is called the BMA [43] method (Bayesian Model Averaging).

With R models $M_1 \ldots M_R$, the posterior probability for $M_k$ given the $x_n$ data is expressed as

$$P(M_k | x_n) = \frac{P(M_k) \int f_k(x_n | \theta_k) \pi_h(\theta_k) d\theta_k}{\sum_{i=1}^{r} P(M_i) \int f_i(x_n | \theta_i) \pi_i(\theta_i) d\theta_i}$$

Marginal likelihoods are estimated using analytical approaches or by performing simulations. In the BMA logic, the predictive distribution z of a future observation $f(z / x_n)$ is defined by

$$f(Z / x_n) = \sum_{j=1}^{r} f(M_j / z) f_j(z / x_n)$$

with $f_j(z / x_n) = \int f_j(z | \theta_j) \pi_j(\theta_j / x_n) d\theta_j \qquad j = 1 \ldots r$

Thus the predictive distribution $f(Z / x_n)$ is the average of the predictive distribution for each model weighted by their corresponding posterior model probability.

When the marginal likelihood can be analytically derived, calculation of the posterior model probabilities $P(M_k / x_n)$, used as weights, may be derived under the assumption that R is sufficiently small. Even when the marginal likelihood cannot be evaluated analytically, Bayesian methods i.e. $P(x_n) = \int f_k(x_n | \theta_k) \pi(\theta_k) d\theta_k$ may be used.

If R is large, calculation may not be possible, in which case the MCMC method can be employed: the MCMC procedure is launched using identical models $\{M_1 \ldots M_R\}$ or reversible jump MCMC.

**B. Reversible jump MCMC model**

The procedure starts with a reversible jump Monte Carlo Markov Chain whose algorithm allows the chain to move over different models. After having defined the jump rules, the posterior probabilities of each model can be directly estimated and thus the marginal likelihood estimation can be avoided.

Let us assume ($M_1 \ldots M_r$) are competing models. The reversible jump MCMC samples over the model and parameter space ($M_k$, $\theta_k$ where k=1…r) generating a Markov chain that can jump from one model to another with parameter

spaces of different dimensions. This algorithm must be reversible, i.e. it must ensure that the chain converges towards a stationary distribution.

If no change in the dimension of the model is involved, the reversible jump MCMC model is constructed on the Metropolis Hastings algorithm. If many Markov chain samples have been generated, the posterior model probability is estimated by $P(M_k | X_n) \approx \frac{1}{L}\sum_{j=1}^{r} 1(M^{(j)} = M_k)$

where L denotes the number of samples and $1(M^{(j)} = M_k) = 1$ if $M^{(j)} = M_k$, else $1(M^{(j)} = M_k) = 0$. In the case of hierarchical and nested models, posterior model probabilities may be compared using a classic likelihood ratio. The BMA method has the advantage of allowing comparison of non-nested models with different dimensions.

### C. Prior for the total population

When information on the whole population is available, this can be used to apply an informative prior. However, assuming we have no information, we then apply Jeffreys' prior for the population, i.e. the inverse informative matrix which is a uniform prior: $p(N) = 1/N$

Since the distribution depends on several parameters (θ), we specify Fisher's information matrix $\{i(\theta)\}$, which is the inverse of the variance-covariance matrix Σ : $\sum = \{i(\theta)\}^{-1}$

Thus Jeffrey's prior for the variance $\sigma^2$ of a parameter is $\pi(\theta) = 1/\sigma$

This is a non-informative, invariant prior.

Alternatively, when prior information is available, we assume that N, with known parameter λ, follows a Poisson distribution $P_o(\lambda)$ i.e.:

$P_o(N = K|\lambda) = e^{-\lambda}\frac{\lambda^K}{K!}$ where λ is the mean of the Poisson distribution

Integrating out the nuisance parameter λ yields N which follows a negative binomial distribution with parameters ($a_1$, $a_2$):

$$P_o(N = K) = \int P_o(N = K, \Delta = \lambda)d\lambda$$

$$= \int P_o(N = K|\Delta = \lambda).P(\Delta = \lambda)d\lambda$$

$$= \int e^{-\lambda}\frac{\lambda^K}{K!}.\Gamma(\lambda)_{a_2, a_2} d\lambda$$

**C.1 Prior for capture rate**

The parameter μ is the capture rate expressed as a logit model (in the absence of any time, behavioral or individual effects). The following prior can be expressed as follows:

$$\mu \sim N(0, 10) \qquad \log\frac{p}{1-p} = 0 \rightarrow \frac{p}{1-p} = 1 \rightarrow p = 1 - P \rightarrow p = \frac{1}{2}$$

However, in the $M_0$ model this does not follow a uniform prior over [0,1] for capture probabilities.

We thus specify a prior with the following probability density function:

$$f(\mu) = e^{\mu} / (1 + e^{\mu})^2$$

i.e. a logistic density function that leads to a uniform prior U [0,1] over the capture probability under model $M_0$.

$$f(\mu) = e^{\mu} / (1 + e^{\mu})^2$$

$$F(S) = \int_{-\infty}^{+\infty} f(\mu) d\mu = \int_{-\infty}^{+\infty} \frac{e^{\mu}}{(1+e^{\mu})^2} d\mu$$

Let $v = e^{\mu}$ and $dv = e^{\mu} d\mu$

$$= \int_{o}^{\infty} dv / (1+v)^2$$

$$= -1 / (1 + v)$$

$$= 1$$

Thus $f(\mu)$ is a density function under **R**

**C.2 Prior for temporal effects**

In ecological surveys, capture rates are often time-dependent.

For each time $\tau = 1, \ldots T$, we define independent priors of the form:

$\alpha_\tau \mid \sigma_\alpha^2 \sim N(0, \sigma_\alpha^2)$ and $\sigma_\alpha^2 \sim \Gamma^{-1}(b_1, b_2)$

where the $\Gamma^{-1}$ distribution is the inverse $\Gamma$ distribution for the mean $b_2 / (b_1 - 1)$ and the variance $b_2^2 / (b_1 - 1)^2 (b_1 - 2)$. Choosing $b_1 = 4$ and $b_2 = 3$ allows to obtain a prior mean and variance with respective values of 1 and 0.5. Thus the corresponding prior distribution for $p_{i\tau}$ is symmetrical around 0.5 and decreases gradually toward values of 0 and 1.

**C.3 Prior for individual effects**

Individual effects $\gamma_i$ are assumed to be random, and the parameters of the distribution of these effects are thus of interest.

In the absence of information relating to these parameters, we specify the following prior:

$\gamma \mid \sigma_\gamma^2 \sim N(0, \sigma_\gamma^2)$ et $\sigma_\gamma^2 \sim \Gamma^{-1}(4, 3)$

This is to be interpreted as a hyperprior on the individual effects where $\sigma_\gamma^2$ is the parameter of interest.

## D. WinBUGS Codes

This $M_{th}$ model thus rests on the assumption that the capture probability p[i,k] is different for each individual *i* (gamma[i]), that it differs between two episodes of capture (alpha[k]). To estimate the total population *N*, using a model that integrates individual heterogeneity, the procedure involves data augmentation [37]. This consists in specifying a theoretical population *M*, with *M*>*N* and representing the upper limit for *N*. Data relating to the captured and therefore

observed population are considered as a data vector x[i] of length $M$ representing the number of T times the individual [i] was captured and is therefore set to 0 for non-captured subjects. The data vector z[i] is assigned the value 1 if the individual is caught and NA (unknown) otherwise.

This model contains more parameters (individual capture probabilities and total population $N$) than observations, which implies applying constraints in order to reach an estimate for N (to provide an inference for $N$). This constraint consists in considering that the individual $p^{mis}$ capture probabilities of the subjects that were not caught are linked to the $p^{obs}$ capture probabilities of those that were captured who provide information on $p^{mis}$. The $p^{mis}$ probabilities are taken into account independently of capture. The $M_h$ model may thus be expressed as $[p^{obs}, p^{mis} | \theta, N]$.

A further difficulty with the $M_h$ model is that the estimated population N is updated at each iteration, and that the dimension of $p$ also varies, which implies creating a loop in WinBUGS, but WinBUGS does not allow a loop index to be stochastic. The WinBUGS code used considers $N$ as the sum of $M$ Bernouilli random variables each with probability φ. Attributing a uniform prior distribution to φ induces a discrete uniform prior distribution for N i.e. {0, 1,…, M}. The vector p [$p^{obs}$, $p^{mis}$] is of dimension M and is updated regardless of whether the individual is captured or not. Another variable z is added to the code, also of dimension M, and equal to 1 if the individual is captured and unknown (NA) otherwise.

```
model{
phi ~ dunif(0,1)
 mu ~ dnorm( 0.0,10)
 sigma2alpha ~ dgamma(3,4)
sigma2gamma ~ dgamma(3,4)

Model~dcat(pi[ ])
for (j in 1:K) {
MI[j]<-equals(Model, j)
}

for (k in 1:3) {
alpha[k]~ dnorm( 0.0,sigma2alpha)

   }
  for( i in 1 : M ) {

gamma[i]~ dnorm( 0.0,sigma2gamma)
      }


            for(i in 1:M){
            for (k in 1:3) {

z[i,k]~dbin(phi,1)

                  logit(p[i,k]) <- mu + b[1]*alpha[k] + b[2]*gamma[i]
                        zp[i,k]<-p[i,k]*z[i,k]
                        x[i,k] ~ dbin(zp[i,k],T)
            }
                  }
for(j in 1:Ncells) {
```

```
b[j]<-Xmod[Model, j]
}

                N<-sum(z[,])
}

list(K=3, Ncells=2,pi=c(0.1, 0.45, 0.45),
Xmod=structure(.Data=c(1, 0,
0, 1,
1, 1),.Dim=c(3,2)),M=850,T=3,x=structure(.Data=c(
#AMHR
1, 1, 1, 1, 1, 1, 1, 1, 1, 1, 1, 1, 1, 1, 1, 1, 1, 1, 1, 1,
1, 1, 1, 1, 1, 1, 1, 1, 1, 1, 1, 1, 1, 1, 1, 1, 1, 1, 1, 1,
…
2, 2, 2, 2, 2, 2, 2, 2, 2, 2, 2, 2, 2, 2, 2, 2, 2, 2, 2, 2,
2, 2, 2, 2, 2, 2, 2, 2, 2, 2, 2, 2, 2, 2, 2, 2, 2, 2, 2, 2,
…
3, 3, 3, 3, 3, 3, 3, 3, 3, 3, 3, 3, 3, 3, 3, 3, 3, 3, 3, 3,
3, 3, 3, 3, 3, 3, 3, 3, 3, 3, 3, 3, 3, 3, 3, 3, 3, 3, 3, 3,
…
0, 0, 0, 0, 0, 0, 0, 0, 0, 0, 0, 0, 0, 0, 0, 0, 0, 0, 0, 0,
0, 0, 0, 0, 0, 0, 0, 0, 0, 0, 0, 0, 0, 0, 0, 0, 0, 0, 0, 0),.Dim=c(850,3))


z=structure(.Data=c(
#AMHR
1, 1, 1, 1, 1, 1, 1, 1, 1, 1, 1, 1, 1, 1, 1, 1, 1, 1, 1, 1,
1, 1, 1, 1, 1, 1, 1, 1, 1, 1, 1, 1, 1, 1, 1, 1, 1, 1, 1, 1,
…
0.5,0.5,0.5,0.5,0.5,0.5,0.5,0.5,0.5,0.5,0.5,0.5,0.5,0.5,0.5,0.5,0.5,0.5,0.5,0.5,
0.5,0.5,0.5,0.5,0.5,0.5,0.5,0.5,0.5,0.5,0.5,0.5,0.5,0.5,0.5,0.5,0.5,0.5,0.5,0.5,
...
0.33,0.33,0.33,0.33,0.33,0.33,0.33,0.33,0.33,0.33,0.33,0.33,0.33,0.33,0.33,0.33,0.33,0.33,0.33,0.33,
0.33,0.33,0.33,0.33,0.33,0.33,0.33,0.33,0.33,0.33,0.33,0.33,0.33,0.33,0.33,0.33,0.33,0.33,0.33,0.33,
...
0, 0, 0, 0, 0, 0, 0, 0, 0, 0, 0, 0, 0, 0, 0, 0, 0, 0, 0, 0,
0, 0, 0, 0, 0, 0, 0, NA, NA, NA, NA, NA, NA, NA, NA, NA, NA, NA, NA, NA,
...
NA, NA, NA, NA, NA, NA, NA, NA, NA, NA, NA, NA, NA, NA, NA, NA, NA, NA, NA, NA),.Dim=c(850,3))
```

The p[i,k] variable is the capture probability for each individual *i* during event k, T is the maximum number of possible captures. The x[i,k] vector of dimension M represents the number of times that the individuals were captured, i.e. 0 for non-captured individuals. To compare results between estimation with 3 episodes of capture and estimation with 21 or 24 capture episodes, we applied the same total number of subjects uncaught (NA) for both of them.

We used codes that allow computation of the Bayesian Model Average [37]. With this algorithm, an estimate for *N* can be obtained that takes all the models into account. The 3 possible $M_{th}$ models are taken into account by multiplying by zero or one the alpha, or gamma components corresponding to the time, and heterogeneity effects, respectively. The Xmod vector allows switching from one model to the other and the variable MI specifies the proportional use of the algorithm for each of the 3 models. The Model variable, defined as the probability vector pi, assigns a prior weight to each model.
